# Etiology of viral induced acute liver failure and defensins as potential therapeutic agents in ALF treatment

**DOI:** 10.3389/fimmu.2023.1153528

**Published:** 2023-04-21

**Authors:** Rafał Hrynkiewicz, Paulina Niedźwiedzka-Rystwej

**Affiliations:** Institute of Biology, University of Szczecin, Szczecin, Poland

**Keywords:** acute liver failure (ALF), defensin, animal model, *Lagovirus europaeus*, rabbit hemorrhagic disease, potential therapeutic agent, antimicrobial peptides (AMPs), rabbit defensins

## Abstract

Acute liver failure (ALF) is a rare and severe disease, which, despite continuous advances in medicine, is still characterized by high mortality (65-85%). Very often, a liver transplant is the only effective treatment for ALF. Despite the implementation of prophylactic vaccinations in the world, the viral background of ALF is still a problem and leads to many deaths. Depending on the cause of ALF, it is sometimes possible to reverse this condition with appropriate therapies, which is why the search for effective antiviral agents seems to be a very desirable direction of research. Defensins, which are our natural antimicrobial peptides, have a very high potential to be used as therapeutic agents for infectious liver diseases. Previous studies on the expression of human defensins have shown that increased expression of human α and β-defensins in HCV and HBV infections is associated with a better response to treatment. Unfortunately, conducting clinical trials for ALF is very difficult due to the severity of the disease and the low incidence, therefore animal models are important for the development of new therapeutic strategies. One of the best animal models that has real reference to research on acute liver failure (ALF) is rabbit hemorrhagic disease in rabbits caused by the *Lagovirus europaeus* virus. So far, there have been no studies on the potential of defensins in rabbits infected with *Lagovirus europaeus* virus.

## 1 Introduction

Acute liver failure (ALF) is a serious consequence of sudden damage to liver cells that can develop over days or weeks and eventually lead to death. Although it is a relatively rare clinical entity, available data indicate an incidence of 1-6 cases per million people per year, accounting for 6% of liver-related deaths and 7% of orthotopic liver transplants in the United States (1). The distinguishing feature of ALF from acute chronic liver failure (ACLF) or decompensated cirrhosis is the absence of pre-existing liver disease. The etiology of ALF is diverse and may include paracetamol toxicity, drug-induced liver injury associated with prescription drugs, herbs and dietary supplements, hepatic ischemia, and autoimmune or viral liver diseases (2). It is estimated that 20-45% of ALF cases have an undetermined cause (2, 3). Despite the high mortality rate, reaching up to 80%, the current state of knowledge about ALF, the availability of treatment methods, including liver transplantation, has improved the prognosis of patients. Currently, liver transplantation is the main treatment method for ALF and remains the only method that enables recovery of patients who have not been cured spontaneously. It is estimated that ALF patients after liver transplantation have a one-year survival rate in the range of 65-70% (4). The incidence of ALF due to viral infection has decreased significantly in Europe, with only 19% of all ALF cases being viral. Despite this, viral hepatitis is still the most common cause of acute liver failure in Asia and Africa in all age groups and in children in Asia and South America (5). For this reason, the search for therapeutic methods of ALF based on antiviral activity is an important and promising direction of research.

Defensins are short antimicrobial peptides (AMPs) present in almost all organisms that are integral to innate immunity. The current state of knowledge about defensins shows that they are characterized by a broad spectrum of antimicrobial activity against bacterial, viral and fungal pathogens, but also exhibit multifaceted immunomodulatory functions. These features of defensins made them a subject of research for the development of therapeutic preparations for infectious diseases (6). Literature data indicate an important role of defensins in ALF-associated virus infections (7). As a result, the development of therapies based on these peptides may prove to be an important research direction that can significantly improve patient survival. However, in the case of clinical entities such as ALF, which are difficult to study clinically due to the rarity of occurrence and high mortality, studies based on animal models are crucial. They enable, above all, a better understanding of pathophysiology, but also the development of potential therapeutic methods (8). One of the proposed animal models of ALF is infection of rabbits (*Oryctolagus cuniculus*) with *Lagovirus (L.) europaeus* virus, which causes severe Rabbit Hemorrhagic Disease (RHD) (9).

In this review, we summarize the key information regarding ALF induced by viral infections, and also shed light on the use of defensins as potential therapeutic agents. Considering the usefulness of the rabbit model of *L. europaeus* infection in research on ALF, we also review the state of knowledge about defensins in rabbits. In this way, we outline the need for further research to use the data obtained in the future to develop therapeutic strategies for the treatment of ALF in humans.

## 2 Viral origin of acute liver failure

Acute liver failure (ALF) is a form of severe liver dysfunction in a patient with no previous history of liver disease. ALF is characterized by severe acute liver injury with encephalopathy in a short time. Defining the root cause of ALF is ambiguous. In developing countries, viral infections (hepatitis A, B and E) are the main cause. In turn, in developed countries, in the United States and most Western European countries, drug-induced damage is considered to be the dominant cause of ALF. This difference results from definitely better sanitary conditions in these countries, as well as the availability of preventive vaccinations (10, 11). In addition to the two most common causes of ALF mentioned above, there are a number of others. Other causes include hypoxia-induced liver damage, acute Budd-Chiari syndrome, veno-occlusive disease, Wilson’s disease, mushroom ingestion, sepsis, autoimmune hepatitis, acute fatty liver of pregnancy, HELLP syndrome (hemolysis, elevated liver enzymes, low platelet count). blood), heatstroke and malignancies (12). The viral background of ALF mainly concerns viral hepatitis infection. The most common virus that causes ALF is HBV (Hepatitis B Virus). ALF due to HBV infection may be the result of acute hepatitis, but also secondary or spontaneous reactivation of HBV in chronic carriers. Several theories have been proposed to explain how ALF is induced in the course of HBV infection (13). Studies have shown (14) that core promoter mutations with a phenotype that increases viral replication are registered in patients with more severe course of the disease and in chronic inflammation. Through increased viral replication, a stronger immune system response and prolonged infection may occur, which may be an important factor leading to ALF (15). An important factor mediating liver damage in HBV infection is the immune response of T lymphocytes (16). However, Farci et al. (17) demonstrated that the onset of ALF in HBV-infected patients was characterized by an overwhelming B-cell response and massive production of IgG and IgM by hepatic lobule-infiltrating plasma cells in the absence of significant T-cell activity. This suggests that B-cell-generated immunity is an important factor in the pathogenesis of ALF in infection HBV and distinguishes this condition from acute hepatitis B (17). An important aspect considered in understanding the substrate of ALF in HBV infection is also the activity of the apoptosis and necroptosis pathway. Gong et al. (18) demonstrated increased activity of these pathways in hepatic failure compared to normal liver by applying bioinformatics strategies, suggesting that these pathways may be central to the pathogenesis of HBV-ALF. HBV infection may be accompanied by co-infection or superinfection with Hepatitis D Virus (HDV). This is because HDV assembly and release depends on the presence of HBV as HDV does not produce its own proteins (19). It is indicated that in the presence of HDV the rate of ALF-HBV approaches 20% in the case of co-infection, and the occurrence of ALF-HBV in the case of superinfection during chronic HBV is also possible (20).

Estimates indicate that HAV (Hepatitis A Virus) accounts for 3% of ALF (15). However, recent studies have shown that HAV is the most common cause of pediatric acute liver failure (PALF) in low- and middle-income countries (21). The prognosis for patients with ALF due to HAV infection is good, with a spontaneous resolution rate of approximately 70%, while the remaining 30% require liver transplantation or death (22). The causality of the development of HAV-ALF is not clear and fully explained. It has been suggested that both the viral agent and host characteristics may influence the likelihood of developing severe disease. Fujiwara et al. (23) reported that nucleotide variations in the central part of the untranslated region (NTR) at the 5’ end of HAV may influence the severity of hepatitis A. On the other hand, age and the presence of concomitant chronic liver disease are important factors that increase the risk of severe infection (24). Vento et al. (25) also reported that in patients with chronic HCV (Hepatitis C Virus) infection, HAV superinfection was associated with an increased risk of acute course and death. There are also indications of a role for immune system components in liver injury, as CD8+ specific T cells have been demonstrated in the liver and blood of patients with acute HAV infection. It has been suggested that CD8+ T cells may lyse infected hepatocytes, but a correlation between the severity of the disease and the level of response mediated by these cells has not been demonstrated so far, therefore the unambiguous role of these cells requires further research (26). Studies in a mouse model have shown that the observed liver damage may result from mitochondrial apoptosis in liver cells as a result of MAVS and IRF3/7 signaling. In addition, classically activated CD8+ T cells, NKT cells, and pro-inflammatory Treg cells were associated with the severity of HAV infection (26–28).

Another important factor of ALF is HEV (Hepatitis E Virus), as it is indicated to be a common cause in Africa and Asia, and accounts for 0.4% of ALF cases in the USA (29). In turn, data from Germany indicate that 10-15% of ALF cases were associated with HEV infection (30). HEV infection is usually self-limiting in healthy patients, but in some cases it can develop into an acute form of the disease, which is observed in pregnant women (31). Additional factors that may affect the development of ALF-HEV are coexisting liver diseases and age (32). Undoubtedly, a very important aspect determining the course of HEV infection is the level of the host’s immune response (33). As the mother’s immune status changes during pregnancy to suit the fetus, this may be a key mechanism for the acute course of infection in pregnant women (34). Comparing women with ALF-HEV with a group of women without ALF, significant differences in TLR expression, inflammatory cytokine production and immune cell compartment were recorded (33). Srivastava et al. (34) also showed that antiviral cellular and humoral responses are related to the severity of HEV infection. In patients with a severe course, an increase in B lymphocytes specific for the HEV antigen, producing IgG antibodies, was recorded. This suggests that progressive liver damage may be mediated by antibodies. In addition, the observed infiltration of CD4+ and CD8+ T cells into the liver in patients with acute HEV infection and acute liver failure in pregnant women may indicate the involvement of these cells in the development of ALF by aggravating inflammatory damage to the liver tissue due to the activation of monocytes and macrophages releasing high levels of pro-inflammatory TNF-α (35).

Data on HCV (Hepatitis C Virus) infection with ALF show only isolated cases of this association. In a study by Rao et al. (36) only three cases out of 2,999 ALF or ALI (Acute Liver Infection) were considered to be related to HCV infection, and all patients had comorbidities. Progressive liver damage in the case of other viral hepatitis is the result of a strong immune reaction of the body. However, HCV eludes immune clearance by suppressing interferon signaling mediators, modulation of complement expression, and neutralizing antibodies, which may explain the rarity of ALF-HCV (37). In addition to viral hepatitis, herpes viruses can also cause ALF, but these cases are relatively less frequently reported. It is indicated that hepatitis caused by Epstein-Barr virus (EBV) infection can lead to ALF with a high mortality rate (38, 39). Thus, EBV is estimated to be responsible for 1.1% of ALF cases in children (40). The mechanism of liver injury in ALF-EBV is not entirely clear, but it is undeniable that liver injury in EBV infection is immunologically mediated by T-cell responses against EBV-infected B cells and CD8+ T-cells infected (41). In the study of Nakajima et al. (42) liver biopsy showed that ALF was due to monoclonal expansion and massive infiltration of EBV-infected CD8+ T cells. Similar observations were recorded by other authors (43, 44) in the case reports of children’s ALF after EBV infection, which confirms the involvement of immune elements in the pathogenesis of ALF-EBV. Herpes simplex virus (HSV)-1 and HSV-2 have also been reported to cause ALF (45, 46). Although rare, ALF-HSV has been reported in neonates, immunocompromised patients and pregnant women. Data indicate that HSV accounts for 0.8% of ALF cases and 1-2% of viral hepatitis (47). Available case reports report that human herpesvirus 6 (HHV-6) infection may also be associated with ALF (48, 49). There are speculations that HHV-6 may influence immunological events which, by disturbing liver homeostasis, lead to the occurrence of ALF (48). There are also isolated reports indicating infection with parvovirus B19 (50), cytomegalovirus (51) and Dengue virus (52) as the cause of ALF.

## 3 Rabbit model of acute liver failure

Acute hepatic failure is associated with a rapid deterioration of the patient’s liver function and is associated with high mortality, which makes it a rather problematic entity for clinical trials. For this reason, research based on various models is a very important direction. Most ALF models are artificial systems, which include surgical, pharmacological or immunogenic models. Immunogenic models include various viral hepatitis models (8). One of them is *Lagovirus europaeus* infection, which causes Rabbit Hemorrhagic Disease (RHD). Terblanche and Hickman (53) described the main criteria that an animal model of acute liver failure should meet: reversibility - treatment will result in the survival of some animals; repeatability – the obtained results can be replicated; the death of the animal occurs as a result of liver failure; therapeutic window; the size of the animal to allow the collection of adequate amounts of blood and tissue for testing; minimal risk to testing personnel. According to Tunon et al. (9) infection with *L. europaeus* virus fulfills all these criteria and is therefore a valid model for ALF. In addition to the changes observed in the liver following infection with *L. europaeus* being consistent with those manifested in humans during ALF, the similarities recorded also apply to physiological, histological and biochemical features (**Table 1**).

**Table 1:** Table showing the similarities between rabbit hemorrhagic disease (RHD) and acute liver failure (ALF) (2, 9, 10, 13, 53–55).

	Rabbit Hemorrhagic Disease (RHD)	Acute Liver Failure (ALF)
clinical features	High mortality rate (80-100%), severe liver failure, multiple organ failure, encephalopathy, disseminated intravascular coagulation (DIC), increase in intracranial pressure (ICP), increase in intracranial pressure, loss of appetite, apathy, anorexia, diarrhea or constipation, neurological changes (agitation, convulsions, paralysis of legs, lateral head rotation, ataxia, coma, apathy), breathing problems, shortness of breath, bloody or bloody foamy discharge from the nose, vaginal hemorrhages, conjunctival hyperaemia and watery eyes, emitting high-pitched sounds, severe jaundice, hyperbilirubinemia, hyperglycemia, coagulopathy (significant decrease in factor V and factor VII activity and deterioration of prothrombin time)	High mortality (65-85%), severe hepatic failure, multiple organ failure, encephalopathy, increased intracranial pressure (ICP), cerebral edema, neuropsychiatric disorders (confusion, disorientation, coma), systemic inflammatory response, body temperature increased, upper right pain parts of the abdomen, bloated abdomen (ascites), nausea, vomiting, drowsiness, breath may have a musty or sweet smell, convulsions, hypoglycaemia, hyperbilirubinemia, jaundice, coagulopathy (decreased factor V activity and prolonged prothrombin time)
biochemical features	Significant increase in biochemical parameters (aspartate aminotransferase (AST), alanine aminotransferase (ALT), gamma-glutamyltransferase (GGT), alkaline phosphatase (ALP), lactate dehydrogenase (LDH), bilirubin, glucose and ammonia), significant increase in the level of amino acids (valine and leucine), decrease in the level of amino acids (arginine), decrease in the Fischer index.	Significant increase in biochemical parameters (aspartate aminotransferase (AST), alanine aminotransferase (ALT), gamma-glutamyl transferase (GGT), alkaline phosphatase (ALP), bilirubin, glucose and ammonia), increase in the level of amino acids (phenylalanine and tyrosine), decrease in the Fischer index.
anatomopathological features	▪ Liver - enlarged with marked lobular pattern, severe disseminated necrotizing inflammation. Changes in the color of the organ to pale, light yellow or gray, and its structure changes to brittle. ▪ Trachea and Lungs - there is hyperemia, exudative inflammation of the mucous membrane, ▪ Heart - changes mainly concern visible focal necrosis and blood extravasation, ▪ Kidneys - swollen, dark red color with visible petechiae, forming a mottled pattern.	▪ Liver - degeneration, severe disseminated necrotizing inflammation, ▪ Lungs - Acute lung damage leading to respiratory distress syndrome in adults, ▪ Heart - the changes mainly concern the visible damage to the heart muscle, ▪ Kidney - Pre-renal damage and internal kidney damage such as tubular necrosis.

Available studies using *L. europaeus* infection as a model for ALF focus on the therapeutic approach. San-Miquel et al. (56) reported that treatment with N-acetylcysteine (NAC) showed hepatoprotective effects in hepatic failure mediated by modulation of the intrinsic apoptosis pathway. Promising results were also obtained by Tunon et al. (57) while analyzing the effect of melatonin on hepatocyte apoptosis.

It was shown that the anti-apoptotic effect of melatonin was dose-dependent and contributed to reduced expression of the pro-apoptotic Bax protein and cytosolic release of cytochrome c, as well as increased expression of the anti-apoptotic proteins Bcl-2 and Bcl-xL and inhibition of the activity of caspase-9, which is a key protein initiating apoptosis. In another study by Crespo et al. (58) showed that melatonin prevented liver damage by inducing significant inhibition of alanine aminotransferase (ALT), aspartate aminotransferase (AST) and lactate dehydrogenase (LDH) and partially abolishing hypoglycemia. In addition, melatonin inhibited oxidative stress and increased the activity of antioxidant defense mechanisms. The therapeutic potential of melatonin was also confirmed by San-Miquel et al. (59). Melatonin was also shown to induce a reduction in autophagy associated with *L. europaeus* infection and inhibited viral replication. In another study by Crespo et al. (60) reported that melatonin, by modulating molecular clock signaling, also inhibited the mitophagy pathway, prevented the activation of the NLRP3 inflammasome, and influenced the innate immune response associated with Toll-like receptor 4 (TLR4) and TLR3, retinoic acid-inducible gene (RIG-1), mitochondrial antiviral signaling (MAVS), natural killer 2D groups (NKG2D) and interferon α (IFN-α), stimulator of interferon (STING), GZMA and perforin (PER1) genes, induced by *L. europaeus* infection.

## 4 Characteristics of defensins

Defensins are a family of cationic peptides of size (2-5kDa), which together with cathelicidins are included in the category of antimicrobial peptides (AMPs). Defensins are produced by the innate immune system and have a broad spectrum of antibacterial, antiviral and antifungal activity (6). They are characterized by a mainly β-sheet structure, which is stabilized by three disulfide bonds. Their diverse structure and organization of disulfide bonds make it possible to divide them into three groups: α-, β- and θ-defensins. Importantly, not all defensins are expressed in every mammal. For example, α-defensins are not expressed in mouse neutrophils, while θ-defensins are only expressed in primates, while β-defensins are widely expressed in mammals (61). Defensins are found in tissues involved in the host’s immune response and are present in leukocyte granules (6). They perform a wide range of regulatory functions in both innate and acquired immunity. In addition to inducing the production of cytokines and chemokines, they also exhibit chemotactic activity against T cells, monocytes and immature dendritic cells (62). Defensins act in innate immunity by depolarizing the cell membrane, opsonizing immune cells, stimulating cytokines and limiting replication by inhibiting nuclear enzymes (62).

**Figure 1:**
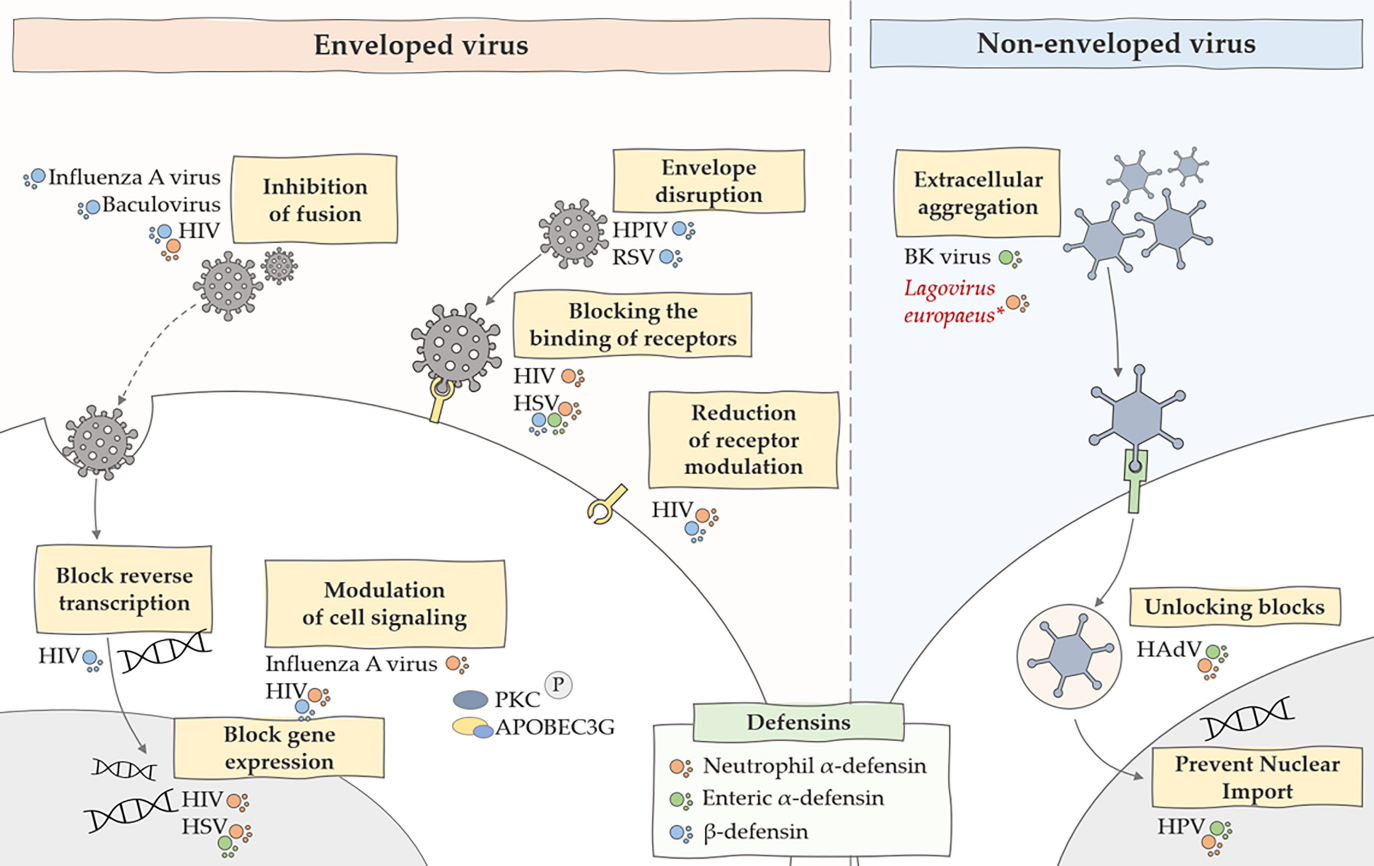
Summary of the main antiviral mechanisms of defensins. The figure shows the inhibitory effect of defensins on many stages of viral infections caused by enveloped and non-enveloped viruses. HIV, human immunodeficiency virus; HSV, herpes simplex virus; HPV, human papillomavirus, HPIV, human parainfluenza viruses, RSV, respiratory syncytial virus; *, predicted effect of rabbit α-defensin on inhibition of *Lagovirus europaeus* infection.

The mechanisms of antiviral action of defensins also focus on direct defensin-virus interaction. They interact with the lipid bilayers of enveloped viruses, and some are also capable of binding to glycoproteins and glycolipids. They can also engage in protein-protein or protein-DNA interactions, and defensin oligomerization and conformational stability can also affect binding. Moreover, the antiviral activity of these peptides is relatively dependent on the type of defensin and virus that we are considering in a given case (63). Defensins also have an inhibitory effect on non-enveloped viruses. Studies have shown (64) that the formation of multimeric forms by defensins can affect virion aggregation. This is because, in addition to the strong binding due to oligomerization, neutralizing the charge of the viral capsid can affect inter-virion repulsion. Such aggregation of viral particles may reduce the level of infectivity by impeding cell binding or cause viral aggregates to enter fewer host cells (64). Other mechanisms of antiviral action of defensins also include inhibition of viral shedding and blocking viral entry to the nucleus of the genome. Some defensins have been shown to inhibit protein kinase C (PKC) signaling, a pathway used by many viruses for fusion, transcription, integration and assembly (65). Moreover, defensins can also activate intracellular antiviral mechanisms related to the CCR6 chemokine receptor and inducing the expression of apolipoprotein B mRNA-editing enzyme, catalytic polypeptide-like 3G (APOBEC3G) (63). A summary of the antiviral effects of defensins is shown in **Figure 1**.

## 5 Defensins in rabbits

**Figure 2:**
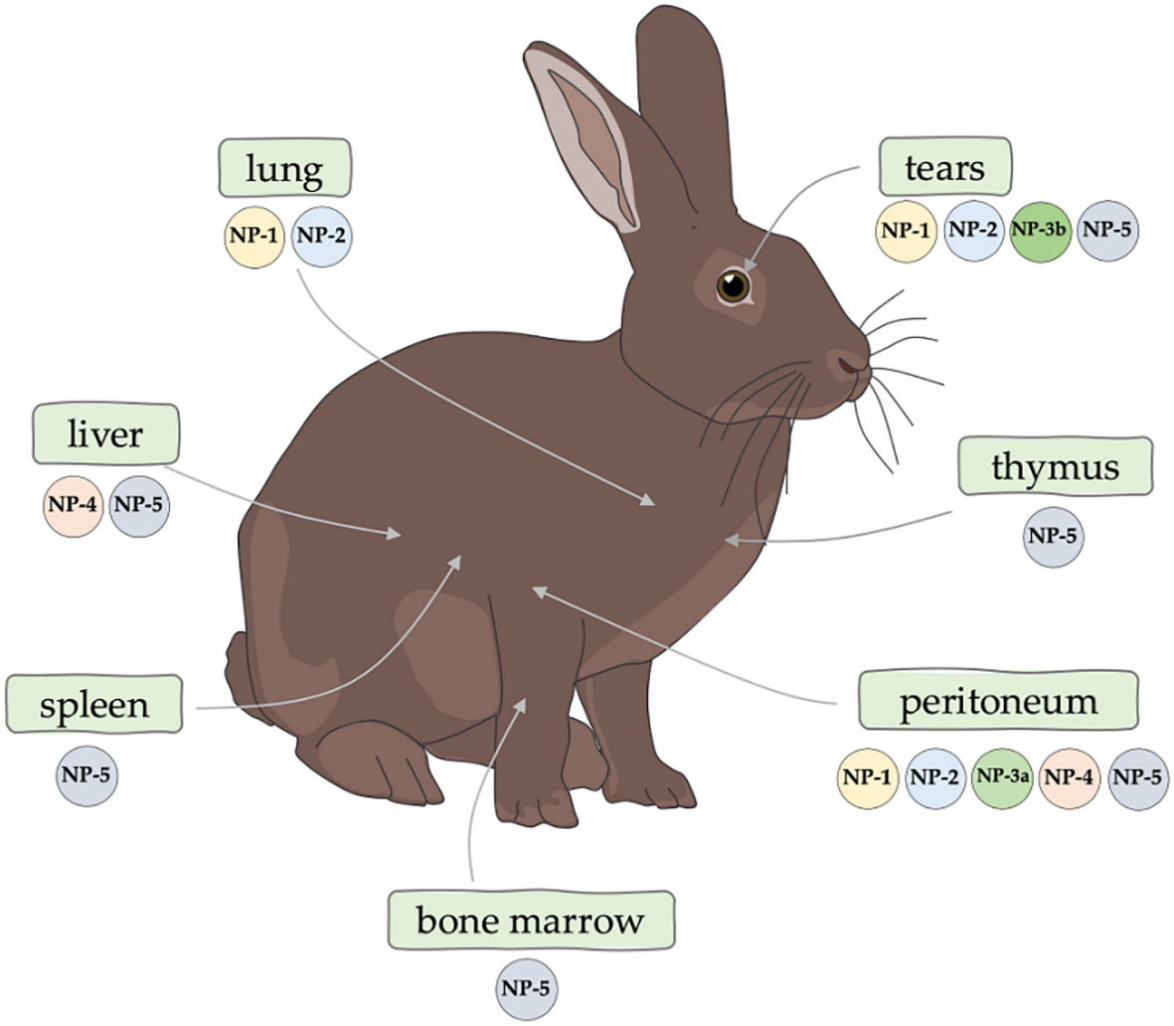
Confirmed occurrence of rabbit α-defensins.

The rabbit is a special organism in the context of defensins. The first mammalian defensin, then called a bactericidal cationic protein, was isolated for the first time from rabbit lung macrophages in 1980 by Lehler et al. (66, 67) in 1985, the same research team isolated peptides from human neutrophils that were homologous to those of rabbits, and then referred to these peptides as “defensins” (68, 69). Currently, six defensins have been described in rabbits (70, 71): NP-1 (otherwise corticostatin-3, antiadrenocorticotropin peptide III, macrophage antibiotic peptide MCP-1), NP-2 (otherwise corticostatin-4, defensin alpha 4, antiadrenocorticotropin peptide IV, macrophage antibiotic peptide MCP-2), NP-3A (otherwise corticostatin-1, antiadrenocorticotropin peptide I, corticostatin-1, microbicidal peptide NP-3A, neutrophil antibiotic peptide NP-3A), NP-3B (otherwise corticostatin-2, antiadrenocorticotropin peptide II, microbicidal peptide NP-3B, neutrophil antibiotic peptide NP-3B), NP-4 (otherwise neutrophil antibiotic peptide NP-4, microbicidal peptide NP-4) and NP-5 (otherwise neutrophil antibiotic peptide NP-5, microbicidal peptide NP-5, corticostatin-6). Defensins are reported to account for at least 15% of the protein content of rabbit neutrophils (72). White et al. (73) demonstrated that rabbit defensins have high sequence and structural similarity to human defensins. Selested et al. (69) determined that rabbit defensins contain 32-34 amino acid residues and are rich in cysteine and arginine, each containing three intramolecular disulfide bonds. The occurrence of defensins in the rabbit body is not fully understood. So far, the presence of defensins (**Figure 2**) in the following sites has been confirmed: NP-1 and NP-2 (peritoneal, lungs, tears), NP-3A (peritoneum), NP-3B (tears), NP-4 (liver, peritoneal) and NP-5 (bone marrow, spleen, thymus, peritoneum, liver, tears) (69, 74–78).

*In vitro* studies have shown that defensin NP-1 has a broad spectrum of antimicrobial activity against bacteria (79, 80), fungi (81), and viruses (82, 83). Hristova et al. (70) studied the interaction of rabbit defensins derived from neutrophils with large unilamellar vesicles (LUVs) made of a mixture of lipids mimicking the lipid composition of Escherichia coli membranes. As a result, it was shown that the defensins NP-1, NP-2, NP-3A, NP-3B and the natural mixture of NP-1-5 induced membrane permeability most likely through transient damage, while NP-4 and NP-5 did not affect membrane permeability. membranes, but had a synergistic effect when used with other defensins. The antiviral functions of defensins were for example analyzed by Sinha et al. (83) in their studies. It was recorded that NP-1 defensin inactivated HSV, prevented virus entry and viral cell-to-cell spread. In turn, Zhou et al. (77) also demonstrated the up-regulation of tear defensin NP-1 and NP-2 during re-epithelialization of an experimental corneal wound. In the study, defensin levels were correlated with the course of wound healing, suggesting an important role for these peptides in protecting the cornea from microbes, as well as a possible involvement in modulating the healing process. Literature data suggest that NP-4 and NP-5 defensins express the weakest antimicrobial properties. However, the effects of NP-5 defensin increase when pathogens are metabolically active (84). Moreover, the antifungal effect of NP-5 defensin was not demonstrated, but its addition in submicromolar concentrations to the solution enhanced the activity of NP-1, NP-2 and NP-3A defensin (85). A summary of the antimicrobial properties of rabbit defensins is provided in **Table 2**.

**Table 2:** Confirmed antibacterial, antifungal and antiviral effect of rabbit α-defensins.

Microorganism	Microorganism	Active defensins	Reference
**Gram positive bacteria**	*Bacillus subtillis*	NP-1, NP-2	(66, 86)
	*Listeria monocytogenes*	NP-1, NP-2, NP-3b	(66, 86, 87)
	*Staphylococcus aureus*	NP-1, NP-2, NP-3a, NP-3b	(68, 86, 88, 89)
	*Staphylococcus epidermidis*	NP-1, NP-2, NP-3a, NP-3b	(86, 90)
	*Streptococcus agalaetiae*	NP-1, NP-2, NP-3a, NP-3b	(86, 90)
	*Streptococcus faecalis*	NP-1, NP-2	(66, 86)
	*Streptococcus pneumoniae*, Type III	NP-1, NP-2, NP-3a, NP-3b, NP-4, NP-5	(86, 90)
**Gram negative bacteria**	*Actinobacillus actinomycetemcomitans*	NP-1	(86, 91)
	*Escherichia coli*	NP-1, NP-2	(66, 68, 86)
	*Haemophilus injluenzae*, Type 3a	NP-1, NP-2, NP-3a, NP-3b, NP-4, NP-5	(86, 90)
	*Pseudomonas aeruginosa*	NP-1, NP-2, NP-3a, NP-3b	(68, 86, 90)
	*Salmonella typhimurium*	NP-1, NP-2	(66, 86, 92)
	*Serratia mareescel1s*	NP-1, NP-2, NP-3a, NP-3b	(86, 90)
	*Treponema pallidum*	NP-2, NP-3a, NP-3b, NP-4, NP-5	(86, 93)
**Fungi**	*Aspergillus fumigatus*	NP-1, NP-4	(94)
	*Candida albicans*	NP-1, NP-2, NP-3a	(84, 86, 87)
	*Coccidioides immitis*	NP-1, NP-2	(86)
	*Cryptococcus neoformans*	NP-1	(86, 95)
	*Rhizopus oryzae*	NP-1, NP-4	(94)
**Virus**	*Herpes simplex virus type 1* (HSV-1)	NP-1	(83, 93)
	*Herpes simplex virus type 2* (HSV-2)	NP-1	(83, 93)

## 6 Defensins – potential therapeutic agent for ALF

Despite significant progress in the development of the pharmaceutical industry and the development of new synthetic and semi-synthetic drugs, drug resistance remains an important problem in the treatment of infectious diseases and is a serious challenge today. For this reason, the search for natural substances with potential therapeutic use is currently a desirable direction of research. Defensins are antimicrobial peptides that are characterized by a wide spectrum of activity, which is why their therapeutic use can bring promising results. Currently, literature data on the role and therapeutic potential of defensins in liver diseases are scarce. Recently, Warner et al. (96) conducted a study on the effects of human β-defensin (HBD-2) on Alcohol-Associated Liver Disease (ALD) in mice. As a result, systematic administration of HBD-2 was shown to attenuate liver damage. In addition, HBD-2 showed immunomodulatory effects by up-regulating cytokines and affecting the abundance of regulatory T cells to create a less pro-inflammatory environment in the liver.

In turn, Zhong et al. (97) reported that knock-out of α-defensins resulted in greater liver damage in a mouse model of alcoholic steatohepatitis, while administration of synthetic human α-defensins 5 showed a therapeutic effect in this study. Mani et al. (98) showed that the level of human β-defensin 1 was significantly elevated in patients with acute or chronic hepatic failure, indicating a strong predictive value as a marker of mortality. There are also reports indicating increased expression of α-defensin 4 in infections with hepatitis viruses, which, as described earlier, are associated with ALF. Zhou et al. (99) showed that the expression of the gene encoding α-defensin 4 (DEFA4) is significantly elevated in patients with acute chronic liver failure (ACLF) associated with HBV. Thus, the involvement of this defensin in the host antiviral response to HBV infection has been suggested. In addition, the authors indicated that the analysis of the expression of this gene may serve as a potential prognostic marker for the detection of ACLF-HBV. Moreover, in another study, β-defensin 3 was upregulated by HBV and prevented intrauterine transmission of HBV to infants (100). Another study showed (101) that DEFA4 expression was elevated in the peripheral blood of HCV-infected liver transplant patients. DEFA4 gene expression analysis was also analyzed in HEV infection (102). As a result, an increased level of expression of α-defensins was recorded in HEV patients, regardless of pregnancy and the clinical picture of the disease, suggesting a protective role of these peptides. The protective role of defensins in HCV infection was also confirmed by Ling et al. (7) in a study on the expression of β-defensin 1 in patients with HCV infection and liver cancer. As a result, it was reported that expression of β-defensin 1 was reduced in HCV-infected liver, and treatment with interferon and ribavirin resulted in increased expression of this defensin. Moreover, the increased upregulation of this expression was associated with a better response to treatment, suggesting a protective role of β-defensin 1 in HCV progression and its predictive value for assessing the effectiveness of interferon and ribavirin treatment (7). The antiviral role of human α and β-defensins and synthetic avian α-defensins was also confirmed by Mattar et al. (103) in an *in vitro* study in tissue culture. The results showed that the defensins used showed strong anti-HCV activity, suggesting that they are potent antiviral agents. The antiviral potential of the defensin BmKDfsin4 derived from the scorpion Mesobuthus martensii Karsch was analyzed in a study by Zeng et al. (104). As a result, BmKDfsin4 was shown to significantly inhibit HBV replication *in vitro* by reducing the production of HBsAg viral core protein. The available data suggest that defensins should be considered as potential therapeutic agents in the treatment of viral infections, and thus of viral ALF.

However, despite the many advantages of using defensins as therapeutic agents, there are also some limitations. These challenges are related primarily to the rapid turnover in the human body, high cost of production or non-specific hemolytic activity on human cells and their immunogenicity. So, more research is required to address these issues (6). Some authors suggest that the use of drugs based on antimicrobial peptides as adjuvants or in combination with other antiviral drugs with different mechanisms of action may prove helpful, which in turn may help to reduce the side effects of treatment and reduce the problem of drug resistance (105).

## 7 Conclusions

Despite the implementation of preventive vaccination in many regions of the world, the viral background of ALF remains a big problem and leads to many deaths (65-85%), therefore the search for effective antiviral drugs seems to be a desirable direction of research. Defensins, being natural antimicrobial peptides, have considerable potential for use as therapeutic agents in infectious liver diseases. Previous studies on the role of human α and β defensins in liver diseases caused by viral infection confirmed the significant role of defensins in the course of the disease. In patients infected with HCV and HBV, a significantly increased expression of defensins is observed, which translates into greater effectiveness of treatment of patients and confirms that defensins are powerful antiviral agents with high therapeutic and prognostic potential in liver diseases. In the case of ALF, conducting clinical trials is quite problematic due to the low incidence and severe course of the disease, due to this fact, studies on the role and therapeutic potential of defensins in liver diseases are scarce, therefore the use of animal models is important for the development of new therapeutic strategies. While searching for an appropriate animal model for ALF, it was observed that the course of rabbit hemorrhagic disease in rabbits is very similar to the course of Acute liver failure. Therefore, experimental infection with *L. europaeus* virus in rabbits is a good and effective model of ALF. For this reason, the role of defensins in infections should be analyzed, and the obtained results may be an important contribution to the development of new therapeutic strategies in infectious liver diseases. So far, there have been no studies on the therapeutic use of defensins in the course of rabbit hemorrhagic disease.

## Author contributions

All authors listed have made a substantial, direct, and intellectual contribution to the work and approved it for publication.
